# Supplementary figures and images for: Optimization of ultrasonic-assisted extraction of polysaccharides and triterpenoids from the medicinal mushroom Ganoderma lucidum and evaluation of their in vitro antioxidant capacities
Source: PLoS One. 2020 Dec 31;15(12):e0244749. doi: 10.1371/journal.pone.0244749 (PMC7774858; doi:10.1371/journal.pone.0244749)

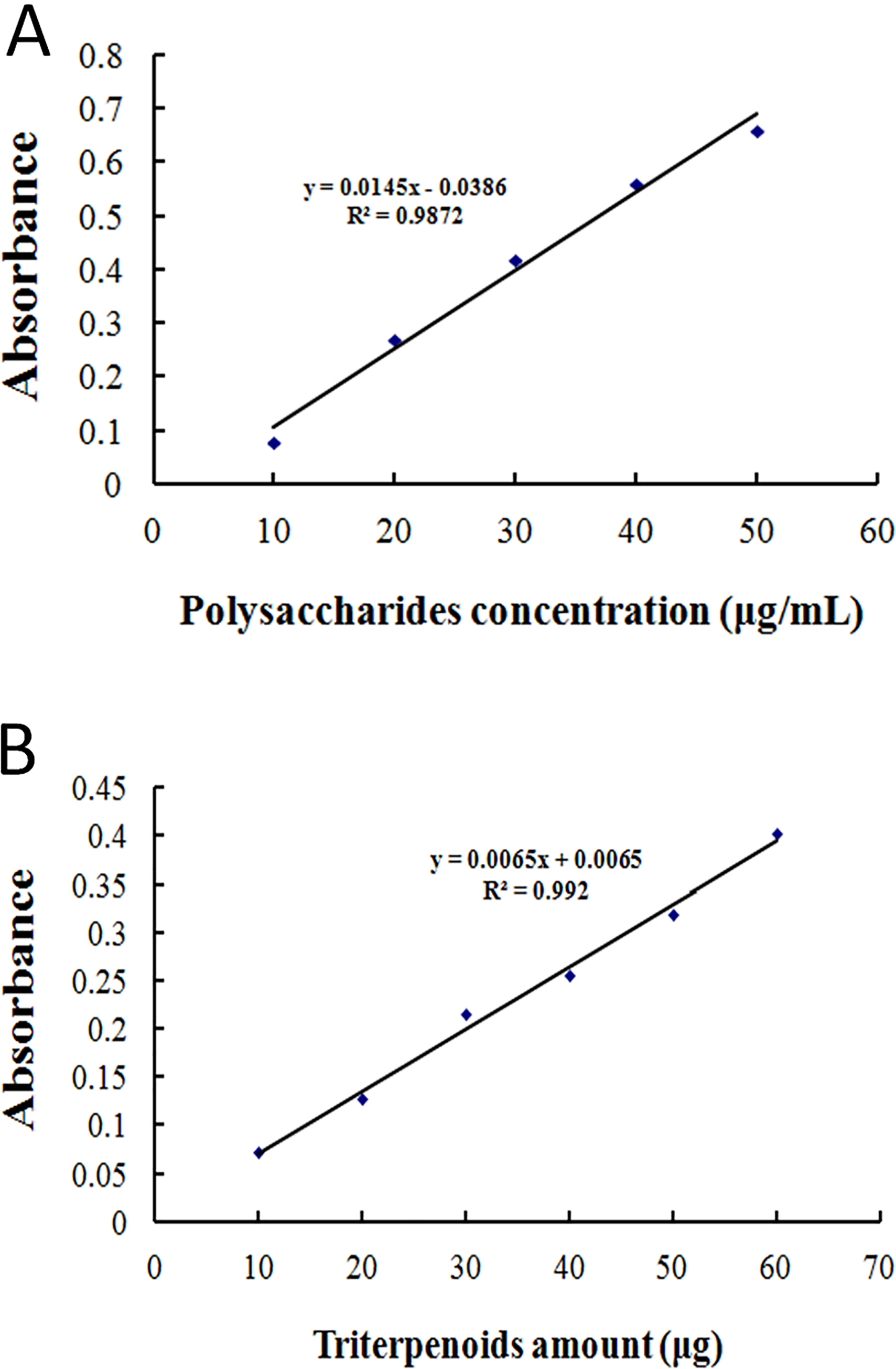

Supplement: S1 Fig — Standard curves for estimation of polysaccharides (A) and triterpenoids (B) used in this work. A, with d-glucose as the standard, and B, with ursolic acid as the standard. (TIF) [file pone.0244749.s001.tif]

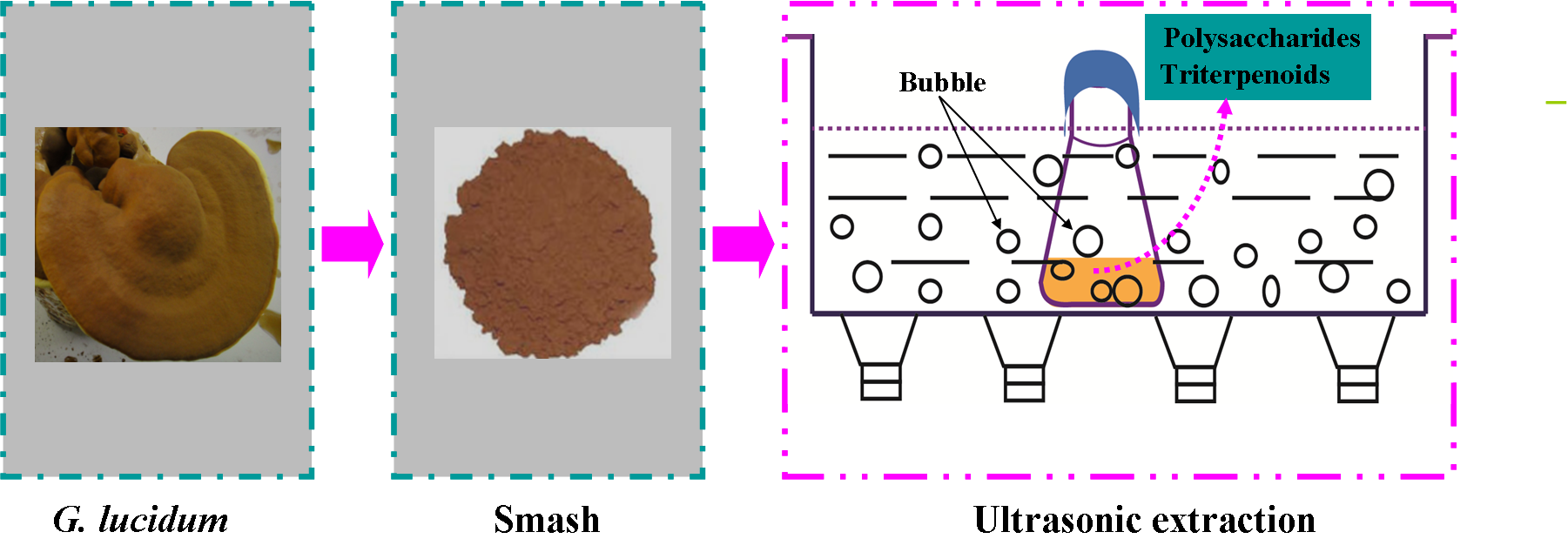

Supplement: S2 Fig — (TIF) [file pone.0244749.s002.tif]
