# Supplementary material for: Optimization of ultrasonic-assisted extraction of polysaccharides and triterpenoids from the medicinal mushroom Ganoderma lucidum and evaluation of their in vitro antioxidant capacities
Source: PLoS One. 2020 Dec 31;15(12):e0244749. doi: 10.1371/journal.pone.0244749 (PMC7774858; doi:10.1371/journal.pone.0244749)
Supplement: Attachment — Submitted filename: Responses to Reviewers.doc [file pone.0244749.s004.doc]

**Responses to the reviewer’s comments**

**Manuscript number:** PONE-D-20-24854

**Title:** Optimization of ultrasonic-assisted extraction of polysaccharides and triterpenoids from the medicinal mushroom *Ganoderma lucidum* and evaluation of their in vitro antioxidant capacities

**Dear Editors,**

We appreciate your comments and suggestions on our manuscript submitted previously. The manuscript has been carefully revised according to the suggestions and recommendation by the reviewers, and now all problems have been addressed in revised manuscript. Revised portion are marked in red in the paper. Point-by-point responses to these comments are showed below in this letter.

Sincerely yours,

Shi-Zhong Zheng, Wei-Rui Zhang, Sheng-Rong Liu

**Reviewer 1#: Some doubts, suggestions and recommendation**

**Comment 1:** Authors have addressed all the major concerns raised in the reviewers’ reports. However, one sentence in the conclusion section fails to present the verity of the study: “The antioxidant activity of the polysaccharides was enhanced by ultrasound”(L480), which does not hold the true since it means that you have somehow extracted polysaccharides, measured their antioxidant activity, let out the ultrasound through them and, measured their antioxidant activity once again and, finally, compared the two values. Please elaborate this.

**Response:** Accept. We have carefully revised this sentence. See in the revision.

**Comment 2:** Additionally, more attention should be paid to the Conclusion section.

**Response:** Accept. We have carefully revised this section, and we believe that the quality was greatly improved. See in the revision.

**Comment 3:** If you would like to make changes to your financial disclosure, please include your updated statement in your cover letter.

**Response:** Thanks. We have updated statement on the financial disclosure in our cover letter.

**List of the changes in revision**

**Abstract**

1. Line 34 of page 2, “crude” was deleted.

2. Line 35 of page 2, “crude” was deleted.

**Introduction**

1. Line 46 of page 3, “The most famous” was revised as “the most highly prized”.

**Results**

1. Line 322 of page 15, “the model was statistically very significant” was revised as “the model was statistically significant”.

2. Line 396 of page 19, “triterpenoids” was revised as “triterpenoid-rich extracts”.

**Discussion**

1. Line 435 of page 20, “in a convenient extraction process” was revised as “in a simple extraction process”.

2. Line 448 of page 21, “have been reported” was revised as “have been reported widely”.

3. Line 458 of page 21, “was shown” was revised as “was also shown”.

4. Line 461 of page 21, “were” was revised as “may be”.

**Conclusion**

1. Line 476 of page 22, “the simultaneous extraction” was revised as “the simultaneous and efficient extraction”.

2. Line 477 of page 22, “from *G*. *lucidum*” was revised as “from the medicinal fungus *G. lucidum*”.

3. Line 477 of page 22, “with ultrasonic technology” was revised as “with ultrasonic extraction technology”.

4. Line 480 of page 22, “On the basis of the scavenging capacity of DPPH and evaluation of reducing power” was inserted.

5. Line 480 of page 22, “The antioxidant activity of the polysaccharides was enhanced by ultrasound” was revised as “the antioxidant activity of the extracted polysaccharides by ultrasonic action in optimal UACE process was significantly enhanced”.

6. Line 480 of page 22, “while the triterpenoid-rich extracts demonstrated unchanged activity” was inserted.
